# RDAR biofilm formation and in silico analysis of putative amyloid proteins in *Azospirillum baldaniorum* Sp245

**DOI:** 10.1007/s00203-026-05297-4

**Published:** 2026-10-31

**Authors:** David Ricardo Cortés-Sotres, María Luisa Xiqui-Vázquez, Sandra Raquel Reyes-Carmona, Nora Georgina Barrera-Olvera, Beatriz E. Baca, Claudia Mancilla-Simbro, Alberto Ramírez-Mata

**Affiliations:** 1 Laboratorio de la Interacción Bacteria-Planta, Centro de Investigaciones en Ciencias Microbiológicas, Instituto de Ciencias, Benemérita Universidad Autónoma de Puebla, Puebla, México; 2 HybridLab. Fisiología y Biología Molecular de Células Excitables, Instituto de Fisiología. Benemérita Universidad Autónoma de Puebla, Puebla, México

**Keywords:** *Azospirillum baldaniorum*, Biofilm formation, Amyloid proteins, RDAR morphotype

## Abstract

*Azospirillum baldaniorum* Sp245 is a plant growth-promoting rhizobacterium (PGPR) whose ability to benefit host crops depends on robust root colonization, a process driven by the transition from a planktonic state to a sessile, biofilm-associated lifestyle. This study investigated the capacity of *A. baldaniorum* Sp245 to form Red, Dry, and Rough (RDAR) morphotype biofilms. Through macroscopic evaluation and confocal laser scanning microscopy (CLSM), we visualized the presence of exopolysaccharides, putative amyloid proteins, and extracellular DNA (eDNA) within the RDAR biofilm matrix using Calcofluor White (CFW), Thioflavin S (TS), and Propidium Iodide (PI) fluorescent probes, respectively. Furthermore, formic acid treatment of extracellular matrix proteins extracts suggests the presence of resistant, putative amyloid proteins. Because genomic screening revealed the absence of homologs of a canonical curli-dependent amyloid system, we performed a genome-wide in silico analysis to identify genes encoding alternative biofilm-associated proteins. Using the *Staphylococcus aureus* Biofilm-Associated Protein (Bap protein) as a reference, we identified 20 candidate proteins characterized by extensive domain reiteration and diverse architectures, including bacterial immunoglobulin (Ig-like) domains, VCBS repeats, SWM motifs, DUF4347, PKD domains, and RTX β-roll structures. Three-dimensional modeling indicates that these candidates possess elongated, filament-like conformations with repetitive β-strand motifs typical of amyloid-like assembly and calcium-dependent adhesion. These findings suggest that in the absence of traditional curli machinery, *A. baldaniorum* Sp245 could be using a diverse, domain-rich repertoire of alternative matrix proteins to facilitate cell surface interactions, coordinate structural stability, and establish the RDAR biofilm morphotype. Ultimately, this study establishes a foundation for understanding the role of amyloid proteins in *Azospirillum* biofilm matrix formation and offers new insights into the potential mechanisms of bacterial persistence and ecological fitness in the rhizosphere.

**Supplementary Information:**

The online version contains supplementary material available at https://doi.org/10.1007/s00203-026-05297-4.

## Introduction

*Azospirillum baldaniorum* Sp245 is a well-studied plant growth-promoting rhizobacterium (PGPR) recognized for its significant contributions to agricultural productivity through multiple mechanisms. These include biological nitrogen fixation and the biosynthesis of phytohormones, such as indole-3-acetic acid (IAA), cytokinins, and gibberellins (Baldani et al. 2015; Cassán et al. 2020; Méndez-Gómez et al. 2021). The capacity of *A. baldaniorum* Sp245 to produce these signaling molecules is well-documented; they modulate host root architecture by stimulating lateral root and root hair proliferation while reducing primary root elongation, thereby increasing root surface area and optimizing nutrient acquisition (Cassán et al. 2020; Nievas et al. 2023). However, the manifestation of these physiological benefits is strictly dependent on robust colonization of the host plant—a process intimately linked to the bacterium’s transition from a planktonic state to a sessile biofilm lifestyle (Salcedo et al. 2015; Mata et al. 2018; Shelud’ko et al. 2025).

In natural environments, the biofilm lifestyle provides a strategic advantage by encasing bacterial communities within a self-produced extracellular matrix. This matrix acts as a structural scaffold and a physicochemical barrier that provides mechanical recalcitrance, nutrient sequestration, and protection against abiotic stressors such as desiccation (Flemming and Wingender 2010). The biofilm matrix is a complex assemblage of extracellular polymeric substances (EPS), including polysaccharides, proteins, lipids, and eDNA, which collectively facilitate adhesion and environmental fitness (Viruega-Góngora et al. 2020; Rath et al. 2025; Shelud’ko et al. 2026).

*Azospirillum* can form biofilms on biotic and abiotic substrates (Viruega-Góngora et al. 2020; Shelud’ko et al. 2025, 2026). Previous studies on the *Azospirillum* biofilm matrix have identified key components required for its construction, including CFW-binding exopolysaccharides, alongside proteinaceous structures such as the polar flagellum, the OmaA adhesin protein, the 67-kDa hemagglutinin (Shelud’ko et al. 2026), and tight adherence (Tad) pili (Burdman et al. 2001; Wisniewski-Dyé et al. 2011; Shelud’ko et al. 2025, 2026). Furthermore, eDNA has been confirmed as a vital constituent for maintaining structural integrity and facilitating surface anchoring (Ramírez-Mata et al. 2016; Viruega-Góngora et al. 2020; Shelud’ko et al. 2026).

Despite the characterization of these matrix components, in vitro research on *Azospirillum* biofilms has been restricted primarily to submerged cultures (liquid-solid interfaces). Consequently, the extracellular matrix composition of biofilms formed at the air-solid interface (agar medium) remains unexplored in this genus. Interestingly, in enteric bacteria such as *Escherichia coli* and *Salmonella enterica* serovar Typhimurium, biofilms developed on agar plates exhibit a distinct macrocolony morphotype known as RDAR (Red, Dry, and Rough). This phenotype is characterized by a colony with a rough, dry surface, net-like warpings, and an undulate margin that spreads over the agar within a few days (Römling et al. 2005; Serra and Hengge 2017). Under the tight regulatory control of the second messenger c-di-GMP, the RDAR phenotype is driven by the coordinated expression of adhesive extracellular components, including cellulose and functional amyloids (FAs) such as curli adhesins (Serra and Hengge 2017; Bu et al. 2005). These matrix elements facilitate surface adhesion, intercellular cohesion, and environmental resilience.

Visualization of the RDAR morphotype is typically enhanced by supplementing agar media with hydrophobic diazo dyes such as Congo Red (CR)—which binds to both cellulose and amyloid fibers—often combined with Coomassie Brilliant Blue (CBB) to aid color discrimination (Römling et al. 2005). In classical models like *E. coli*, biofilm-producing strains express cellulose and *curli* fibers as core matrix components (Römling et al. 2005; Milanov et al. 2015).

Milanov et al. (2015) demonstrated that strains producing the RDAR phenotype on CR agar plates co-express both *curli* and cellulose, whereas PDAR (pink, dry, and rough) phenotype expresses only cellulose, BDAR (brown, dry, and rough) expresses only *curli*, and the SAW (smooth and white) phenotype lacks both. Further variations, such as RAS (red and smooth) or PAS (pink and smooth), reflect exclusive cellulose production, while BAS (brown and smooth) triggers solely *curli* (Bokranz et al. 2005).

Building on the capacity of CR and other dyes to bind diverse EPS components, macrocolony assays have been successfully adapted to characterize matrix architectures in other non-enteric models, such as *Bacillus subtilis* (e.g., the amyloid TasA) and *Pseudomonas aeruginosa* (e.g., PEL and PSL polysaccharides) (Römling et al. 2005). Moreover, this type of biofilm model has facilitated the characterization of genes participating in c-di-GMP metabolism and exopolysaccharide synthesis, as well as the identification of functional amyloids (Herbst et al. 2015; Limoli et al. 2015; He et al. 2018; Pérez-Mendoza et al. 2026).

However, unlike enteric bacteria that rely specifically on the *csg* operon for *curli* assembly, the PGPR *Azospirillum* lacks curli-encoding genes. Nevertheless, the RDAR morphotype serves as a critical, universally conserved model for understanding how bacteria transition from single-cell states into protected, multicellular communities capable of resisting harsh environmental challenges (Römling et al. 2005).

This lack of knowledge regarding air-solid interface communities on agar plates and the components comprising their extracellular matrix in *A. baldaniorum* Sp245 represents a significant gap in our understanding of how *A. baldaniorum* maintains ecological fitness and persistent colonization under fluctuating environmental conditions.

In this work, we investigated the development of the RDAR-type biofilm in *A. baldaniorum* Sp245. To dissect the composition of extracellular matrix (ECM), we utilized CR and CBB on agar plates. Subsequently, we visualized the presence of matrix components using different fluorophores coupled with CLSM via the inverted agar method: CFW for exopolysaccharides, TS for functional amyloids, and PI for eDNA. The fluorescent signals from CFW and PI suggest la presence of exopolysaccharides, and eDNA, respectively. Furthermore, macroscopic staining with CR and TS, microscopic detection with TS, and the resistance of ECM extract proteins to formic acid treatment collectively indicate the presence of putative functional amyloids within the biofilm matrix, despite the absence of classical curli-coding genes.

Building upon these experimental findings, we conducted a genome-wide in silico analysis of *A. baldaniorum* Sp245 to identify candidate genes encoding amyloidogenic proteins that mediate structural assembly. Together, these approaches and findings establishing a foundation for future studies exploring the spatial regulation of matrix components that driving multicellular biofilm construction in *A. baldaniorum* Sp245.

## Materials and methods

### Bacterial strain growth conditions and Congo Red biofilm assay

*A. baldaniorum* Sp245 strain (Baldani et al. 1987) was maintained on Congo red medium and cultures for biofilm assays on modified Lysogeny Broth (LB*) agar without salt: 10 g/L Tryptone, 5 g/L yeast extract, CaCl_2_. 3.67 g/L, MgSO_4_ 6.16 g/L and 15 g/L Agar for assays. To assess the growth of bacterial macrocolonies for RDAR morphotype development, cells were grown overnight in liquid K-malate medium with shaking (120 rpm) at 30 °C. The overnight cultures were grown to an optical density at 600 nm (OD_600_) of 1.1–1.4 and subsequently adjusted to an OD_600_ of 2.0 in NaCl-free modified LB medium. Then, 10 µL aliquots were spotted onto NaCl-free modified LB agar plates supplemented with Congo red (40 µg /mL, Sigma-Aldrich™) and Coomassie Brilliant Blue (20 µg/mL, Merk™), or CFW (85 µM, Sigma-Aldrich™), TS (40 µg/mL, Sigma-Aldrich™), and PI (0.1 mM, Sigma Aldrich. The strain was grown on the agar plates and incubated at 30 °C for 5 days. Macrocolony images were captured using a digital camera and, when required, under UV-A light (315–400 nm wavelength range) using a documentation system (Bio-Rad).

### Analysis of the *A. baldaniorum* macrocolony matrix components by CLSM

To characterize the distribution of exopolysaccharides (EPS), putative functional amyloids, and extracellular DNA, *Azospirillum* macrocolonies were visualized using CLSM in conjunction with the inverted agar method (Hickey and Read 2009). Briefly, *A. baldaniorum* Sp245 was cultured on NaCl-free modified LB agar supplemented with CFW; 85 µM, TS; 40 µg/mL and PI; 0.1 mM. Following five days of incubation at 30 °C, the RDAR macrocolonies were obtained intact by excising the underlying agar and carefully transferring them onto 24 × 50 mm glass coverslips. This procedure effectively creates a “sandwich” configuration positioning the macrocolony between the glass coverslip (bottom) and the agar layer (top) facilitating high resolution imaging without disrupting the biofilm architecture.

Confocal images were acquired using a Nikon C2 + CLSM (Nikon, Tokyo, Japan) equipped with CFI Plan Apo Lambda 10x and 60x objectives. Fluorescence was elicited using an argon laser at specific excitation and emission wavelengths: CFW (Excitation λ = 350 nm; Emission λ = 432 nm) and TS (Excitation λ = 391 nm; Emission λ = 428 nm), and PI (Excitation λ = 535 nm; Emission λ = 617 nm).

### Fluorescence microscopy

RDAR macrocolonies were cultured on NaCl-free modified LB agar supplemented with TS for 5 days at 30 °C. To prepare samples for fluorescence microscopy, material from various macrocolony regions (from the center to periphery) was harvested using a pipette tip and responded in a 12 µL drop of sterile distilled water on a 24 mm x 50 mm coverslip. The suspension was stained with 1.5 µL of the lipophilic fluorescent dye FM4-64 (10 µg/mL) (Thermo Fisher Scientific) and covered with a thin 1% agarose pad. Samples were observed using a Nikon Eclipse TE2000-U fluorescence microscope equipped with 100x objective. Fluorescence was excited/emitted at 565/734 nm for FM4-64 and 391/428 nm for TS. We used as control planktonic bacteria to determine no signal of TS in this stage of growth in *A. baldaniorum* Sp245.

### Biofilm extracellular matrix (ECM) extraction and formic acid treatment assay

*Azospirillum* macrocolony biofilms were grown in K-malate liquid medium and incubated overnight at 30 °C with shaking. The overnight cultures were adjusted to an optical density (OD _600_) of 1.1–1.4 and subsequently adjusted to an optical density of 2.0 in NaCl-free modified LB medium. Then, 10 µL aliquots were spotted onto NaCl-free modified LB agar medium, and the plates were incubated statically at 30 °C for 5 days. Following incubation, the colony biofilms were scraped using a bacteriological loop and suspended in 0.3 mL of 1.5 M NaCl solution. The suspension was incubated for 10 min at room temperature and subsequently centrifuged at 5000 x g for 10 min at room temperature. The resulting supernatants were collected as the ECM fractions.

To analyze potential protein amyloids within the extracted ECM fractions, the samples were dialyzed using Amicon centrifugal filter units according to the manufacturer’s recommendations and resuspended in 16 mM Tris-HCl. The dialyzed extract was divided into three equal fractions: the first fraction received no treatment; the second fraction was treated with 2.0% sodium dodecyl sulfate (SDS); and the third fraction was treated with 2.0% SDS and 5 µL of 99% formic acid. Subsequently, the samples were subjected to SDS-PAGE, and following electrophoresis, the gels were stained with CBB.

### Bioinformatic analyses

To identify potential biofilm associated proteins, typical bioinformatic approaches were used, including the following: *(i) Gene Annotation*: Genome sequencing data analyzed to identify large proteins that are predicted to be involved in biofilm formation. *(ii) Domain identification*: Biofilm-associated proteins with amyloid-related structures usually contain specific functional domains, the SMART protein platform was used to predict functional domains and protein architectures based on the aminoacidic sequence of the protein (Schultz 2000). *(iii) Bap-like and/or FA potential proteins aminoacidic sequence search.* To identify genes encoding potential Bap proteins in the genome of *A. baldaniorum* Sp245, a BLAST search was conducted using the amino acid sequence of a previously reported Bap from *Staphylococcus aureus* V329 (Accession number AAK38834.2), annotated as a biofilm-associated surface protein. *(iv) Protein Modelling*,* Molecular Conservation*,* and Structural Analysis*. The amino acid sequences of the proteins identified as potential Bap from the *A. baldaniorum* Sp245 genome were submitted to AlphaFold3 for protein structure prediction. The predicted 3D structures were downloaded in mmCIF format (.cif) and analyzed using ChimeraX software (version 1.6) (Pettersen et al. 2021). Predicted protein models were compared to previously characterized amyloid structures to assess structural similarity and potential amyloidogenic features.

## Results

**Fig. 1:**
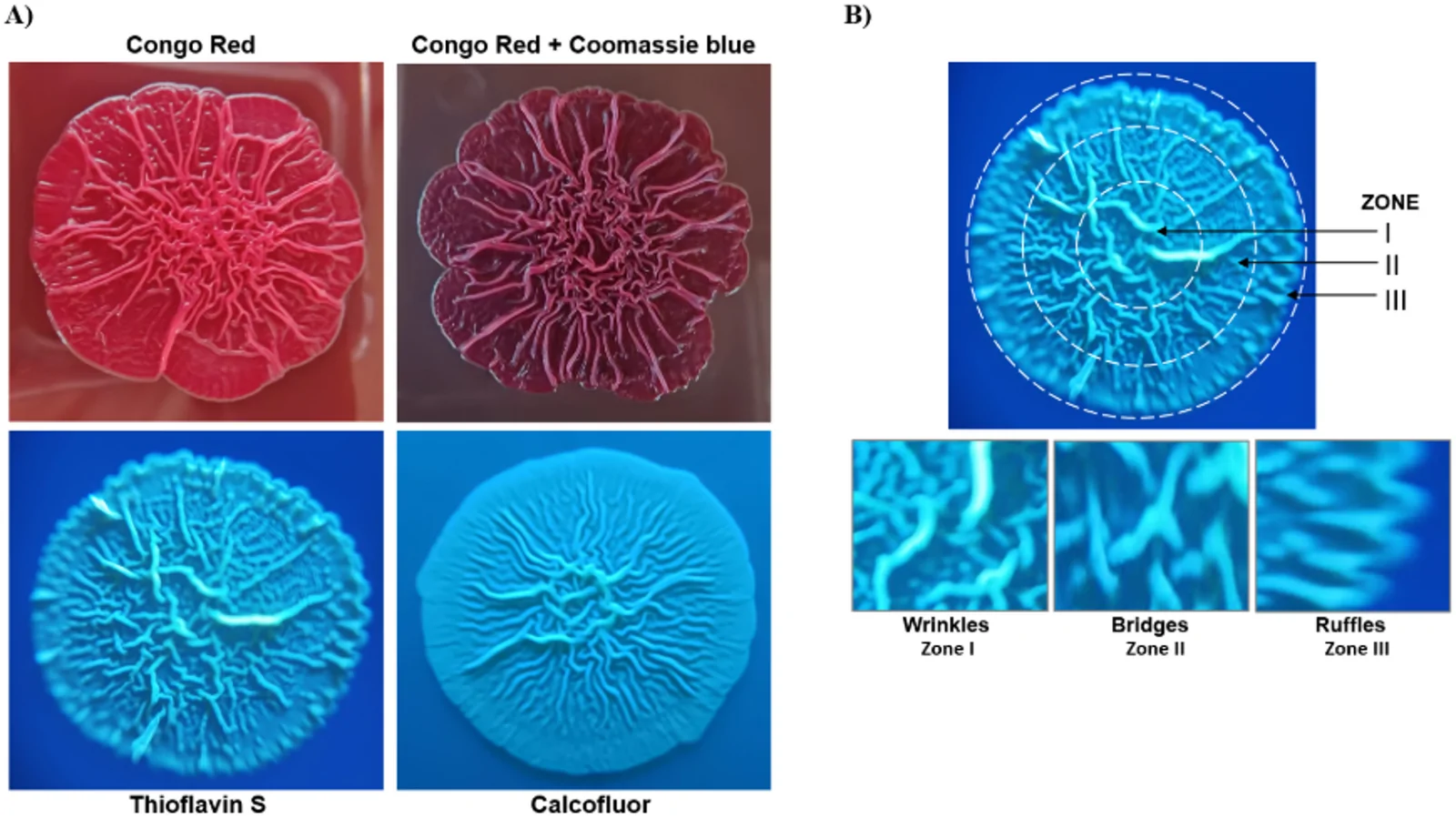
RDAR morphotype in *A. baldaniorum* Sp245. **A** Wild-type macrocolonies grown on NaCl-free LB* agar plates for 5 days at 30 °C, supplemented with Congo red, Congo red/Coomassie Brilliant Blue. CFW, or TS. Images are representative of three technical replicates. **B** Structural zones of the RDAR morphotype within the macrocolony. Zone I represent the highly structured central region characterized by wrinkles; Zone II is characterized by bridge-like structures; and Zone III (the outer ring) consists of ruffles. These morphological features are consistent with the RDAR phenotype previously described (Serra and Hengge 2017)

In this study, we analyzed the capacity of *A. baldaniorum* Sp245 to form biofilms at the air-solid interface on agar plates, observing a behavior resembling the RDAR (Red, Dry, and Rough) morphotype previously reported in *Enterobacteriaceae*. Specifically, when grown on NaCl-free LB medium supplemented with CR, the macrocolonies appeared red, dry, and rough after 5 days of culture (Fig. 1A). These macrocolonies display a rugose architecture organized into three distinct concentric zones (I, II, and III), reflecting the developmental patterns reported in *Enterobacteriaceae* (Römling et al. 2005; Cimdins and Simm 2017; Serra and Hengge 2017): Zone I (Center) features a dense network of randomly oriented, bridge-like wrinkles; Zone II (Intermediate) exhibits prominent, radially oriented bridge-like structures; and Zone III (Outer Ring) constitutes a narrow peripheral region largely devoid of complex architecture, exhibiting instead ruffled structures (Fig. 1B).

Furthermore, when CR was combined with CBB, the colonies turned brown, dry, and rough, suggesting an increased protein abundance (Fig. 1A). To analyze the extracellular matrix composition, we initially employed CFW; for exopolysaccharides and TS; for putative functional amyloids separately. Neither fluorophore inhibited the RDAR phenotype; macrocolonies retained their dry, rough appearance while exhibiting good fluorescence, indicating the presence of CFW-binding exopolysaccharides and TS-binding proteins. Combining these staining results suggests that the *Azospirillum* RDAR morphotype contains both CFW-staining exopolysaccharides and proteins (Fig. 1A). These proteins could be of the amyloid type, alongside others previously detected in *Azospirillum* biofilms, on liquid media, such as polar flagellin, OmaA protein, hemagglutinin, and the TAD pilus (Viruega-Góngora et al. 2020; Shelud’ko et al. 2026).

**Fig. 2:**
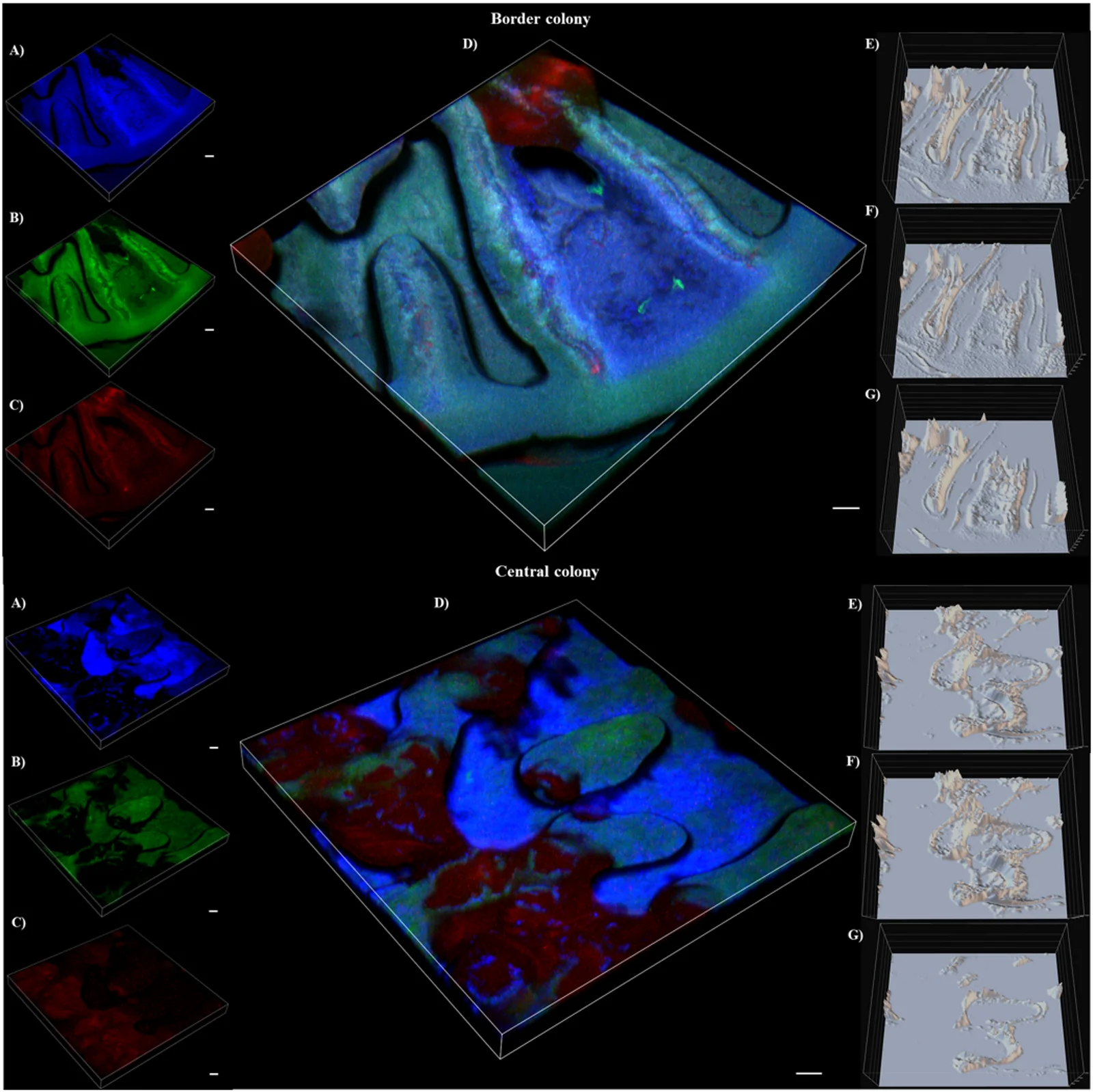
CLSM images of the RDAR type biofilm of *A. baldaniorum* Sp245. Upper panel. Wild type strain macrocolonies grown on NaCl-free modified LB agar for 5 days at 30 °C were observed at the CLSM microscope and images obtained are shown in border zone acquired with 10x objective. The images are representative of three independent experiments with three technical replicates. Images show the spatial distribution of the biofilm components stained by means of fluorescent dyes. **A** CFW (exopolysaccharides; blue). **B** TS (Amyloids; green) and **C** PI (eDNA; red). **D** Merge of three fluorescent channels. **E**, **F** and **G** representative intensity surface plots of each fluorescent channels. Lower panel corresponds to central colony zone. **H** CFW (exopolysaccharides; blue). **I** TS (Amyloids; green) and **J** PI (eDNA; red). **K** Merge of three fluorescent channels. **L**, **M** and **N** representative intensity surface plots of each fluorescent channels. Scale Bar 50 μm

**Fig. 3:**
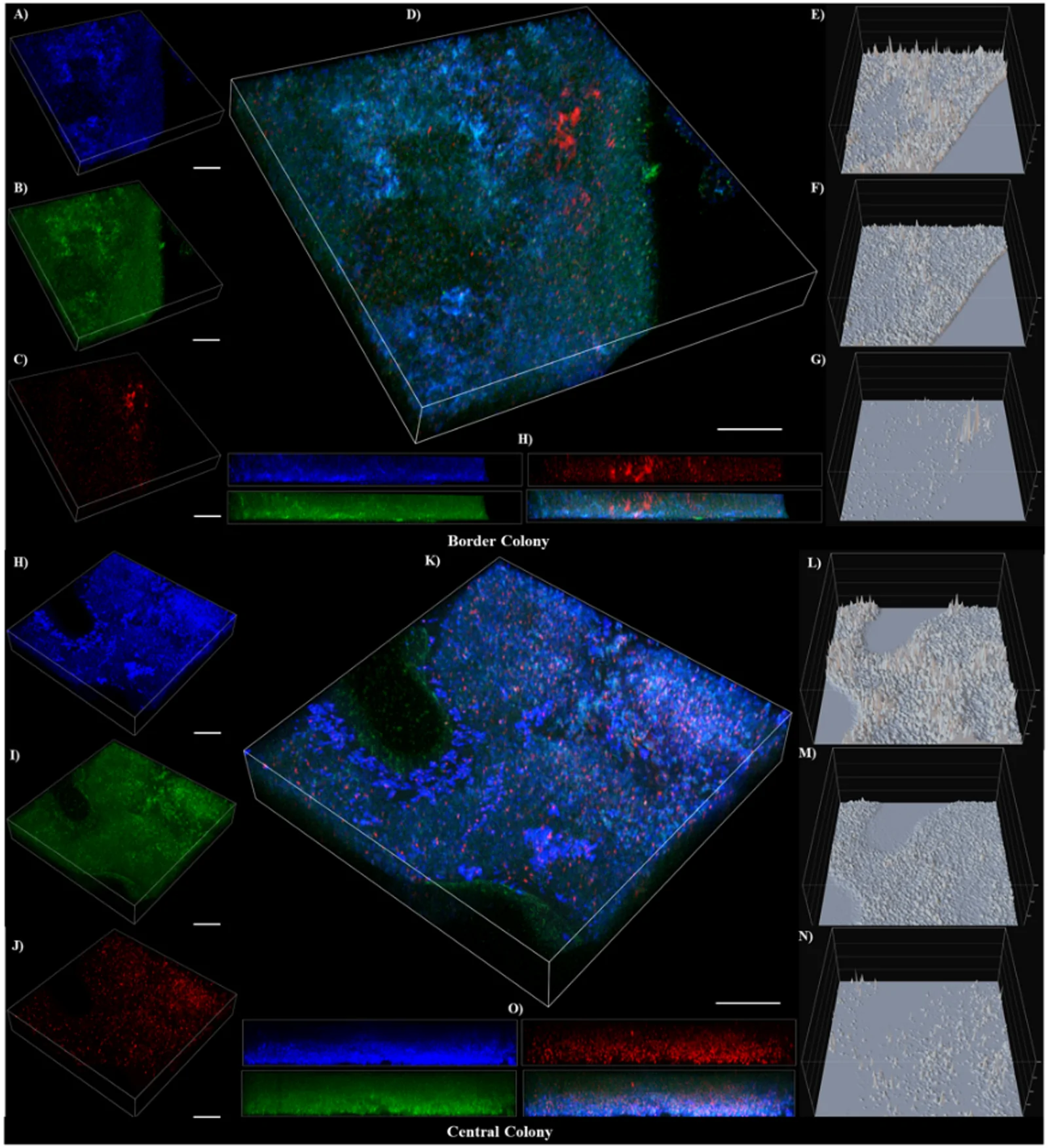
High-resolution 3D visualization of the *A. baldaniorum* Sp245 RDAR-type biofilm. Upper panel. Wild-type strain macrocolonies were grown on NaCl-free modified LB agar for 5 days at 30 °C. CLSM images were acquired using a 60x objective from the border zone of the biofilm. Data are representative of three independent experiments with three technical replicates. 3D projections depict the spatial distribution of biofilm components stained with fluorescent dyes: **A** CFW (blue); **B** TS (green); and **C** PI (red); **D** Merged image of all three channels. The observed TS fluorescence suggest the presence of putative amyloid proteins in the *A. baldaniorum* biofilm. **E–G** Representative intensity surface plots for each fluorescent channel. **H** Orthogonal (axial) views of each fluorescent channel. Scale Bar 50 μm. Lower panel corresponds to central colony zone. **I**) CFW (blue). **J** TS (green) and **K** PI (red). **L** Merge of three fluorescent channels. **M**, **N** and **O** representative intensity surface plots of each fluorescent channels. Scale Bar 50 μm

**Fig. 4:**
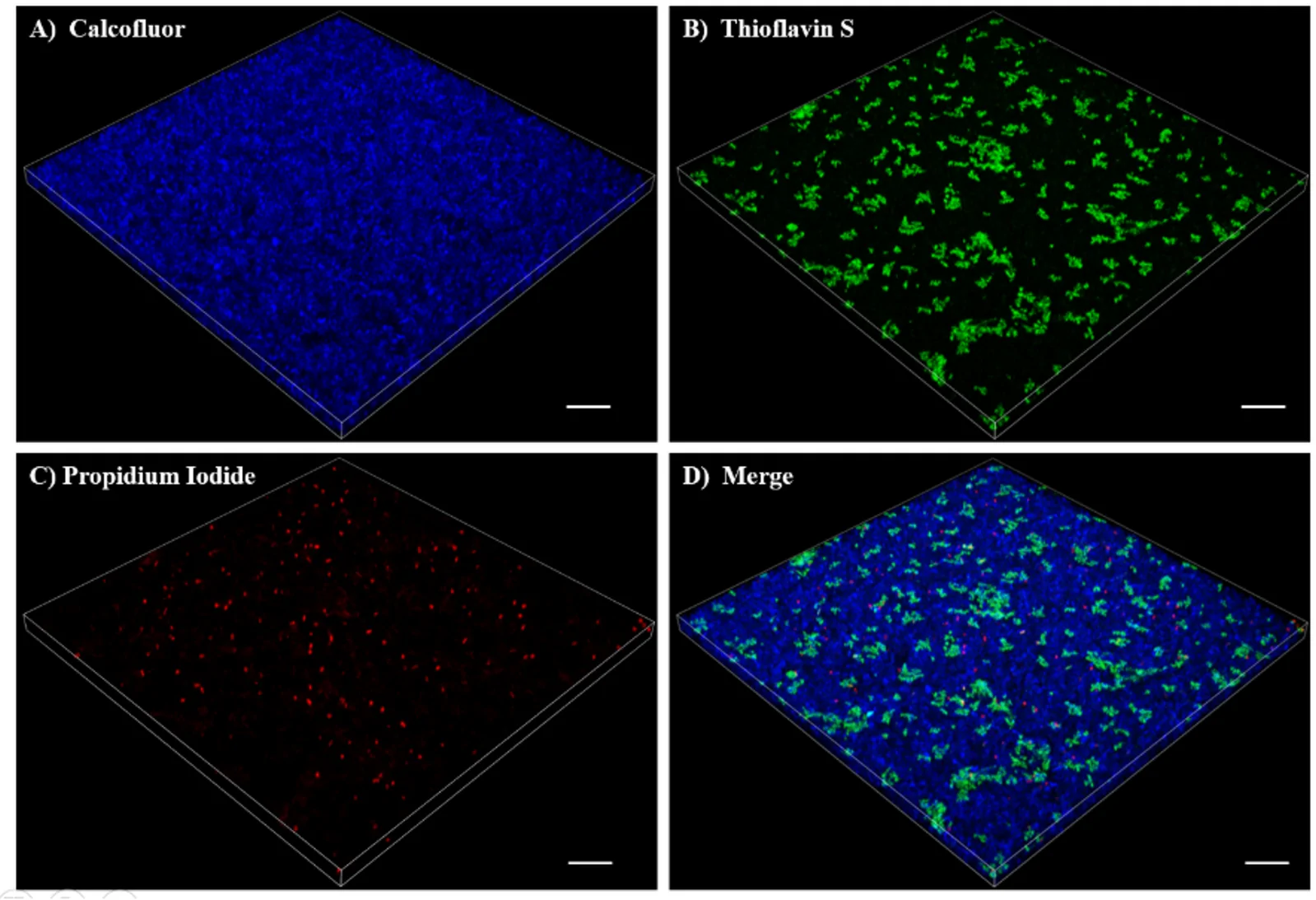
High-resolution 3D visualization of the *A. baldaniorum* Sp245 RDAR-type biofilm. Wild-type strain macrocolonies were grown on NaCl-free modified LB agar for 5 days at 30 °C. CLSM images were acquired using a 60x objective from the central zone of the biofilm, with 1.5 digital zoom. Data are representative of three independent experiments with three technical replicates. 3D projections depict the spatial distribution of biofilm components stained with fluorescent dyes: **A** CFW, exopolysaccharides (blue); **B** TS, amyloids (green); and **C** PI, eDNA (red). **D** Merged image of all three channels. The observed TS fluorescence suggest the presence of putative amyloid proteins in the *A. baldaniorum* biofilm

To delve deeper into the matrix components, we examined macrocolonies using Confocal Laser Scanning Microscopy (CLSM) with 10 x and 60 x objectives (Figs. 2 and 3). We observed abundant EPS across the macrocolony, characterized by blue fluorescence from CFW-binding exopolysaccharides, green signals indicative of putative functional amyloids, and other type of proteins, red fluorescence corresponding to eDNA or cells with compromised membranes. To better visualize the fluorescent signal of each fluorophore, we generated three-dimensional reconstructions and timelapse captures using the 60x objective with a 1.5 x optical zoom. As shown in Fig. 4 and Video 1, the fluorophores successfully detected and localized the various components participating in the construction of the biofilm extracellular matrix.

**Fig. 5:**
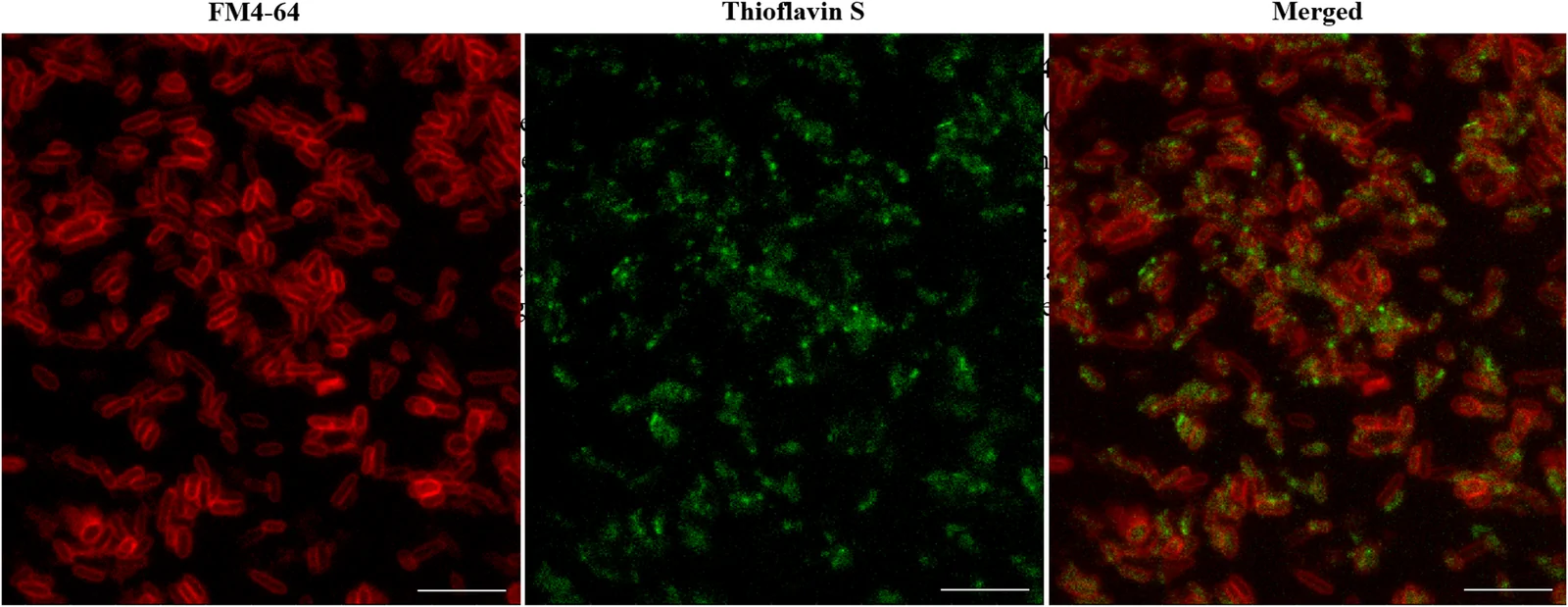
Fluorescence microscopy analysis of the *A. baldaniorum* Sp245 RDAR morphotype. Representative images of 5 days old biofilms grown on NaCl-free modified LB agar supplemented with FM4-64 (red) and Thioflavin S (green) fluorophores. Images were acquired using a 100x objective. The scale bar represents 10 μm

**Fig. 6:**
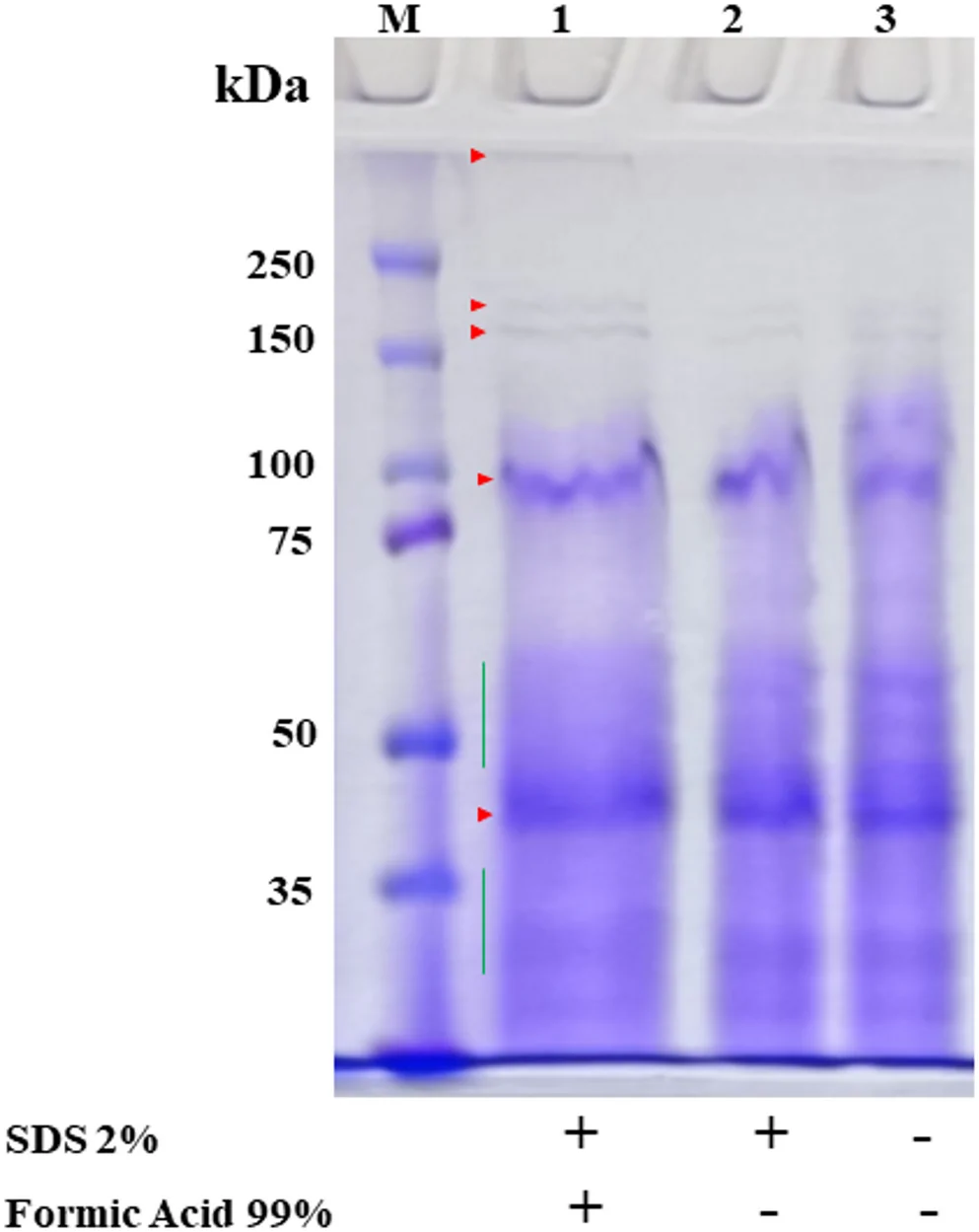
Extracellular matrices of *A. baldaniorum* Sp245 were isolated with 1.5 M NaCl. ECM protein fractions were treated with or without, SDS or formic acid (FA). and analyzed by SDS–polyacrylamide gel electrophoresis (SDS-PAGE). Lanes 1 and 2 contain ECM protein extracts treated with and without FA, respectively. Lane 3 contains untreated ECM protein extract. Lane M Molecular weight markers (sizes in kilodaltons are indicated on the left). Gel was stained with CBB. Red tringles indicate formic acid resistant proteins, whereas green lines highlight degraded proteins

Supplemental fluorescence microscopy (100x objective) using FM4-64 (for membrane phospholipid detection) and TS suggests the presence of putative functional amyloids on the cell surface (Fig. 5). To further investigate these probable amyloid proteins, present in the ECM, we followed the extraction protocol reported by (Chiba et al. 2015), and treated the samples with formic acid, exploiting the characteristic resistance of amyloid proteins to this treatment (Chapman et al. 2002). As shown in the Fig. 6, this analysis suggests the presence of amyloid-type proteins in this biofilm, which will be further analyzed, and identified in future studies using mass spectrometry.

Collectively, these findings indicate that proteins-including functional amyloids- alongside exopolysaccharides, and eDNA, constitute the extracellular matrix (ECM) of the colonial morphotype; however, further functional analyses are necessary to definitively validate their specific roles in the RDAR phenotype.

### In silico prediction and structural analysis of biofilm-associated proteins in *A. baldaniorum* Sp245

Because there are no reports to date of amyloid-like proteins identified in *Azospirillum* strains, and given that this and previous studies suggest the presence of amyloids proteins (Shelud’ko et al. 2018), we performed a bioinformatic analysis of the complete genome sequence of *A. baldaniorum* Sp245. Since extensive literature indicates that Biofilm-associated proteins (Baps) and curli fimbriae amyloids are critical components of the extracellular matrix and are closely linked to the RDAR morphotype in various species (Cimdins-Ahne et al. 2023 a; Olubajo and Tükel 2024), we screened the genome, using the *E. coli* curli amino acid sequence (WP_000771435.1) and the *Staphylococcus aureus* V329 Bap protein (AAK38834.2) as queries (Figure S1). This screening revealed no homologs of CsgA or the *csgBAC-csgDEFG* operons, indicating that *A. baldaniorum* Sp245 lacks a canonical curli-dependent amyloid system (Figures S2 and S3).

**Fig. 7:**
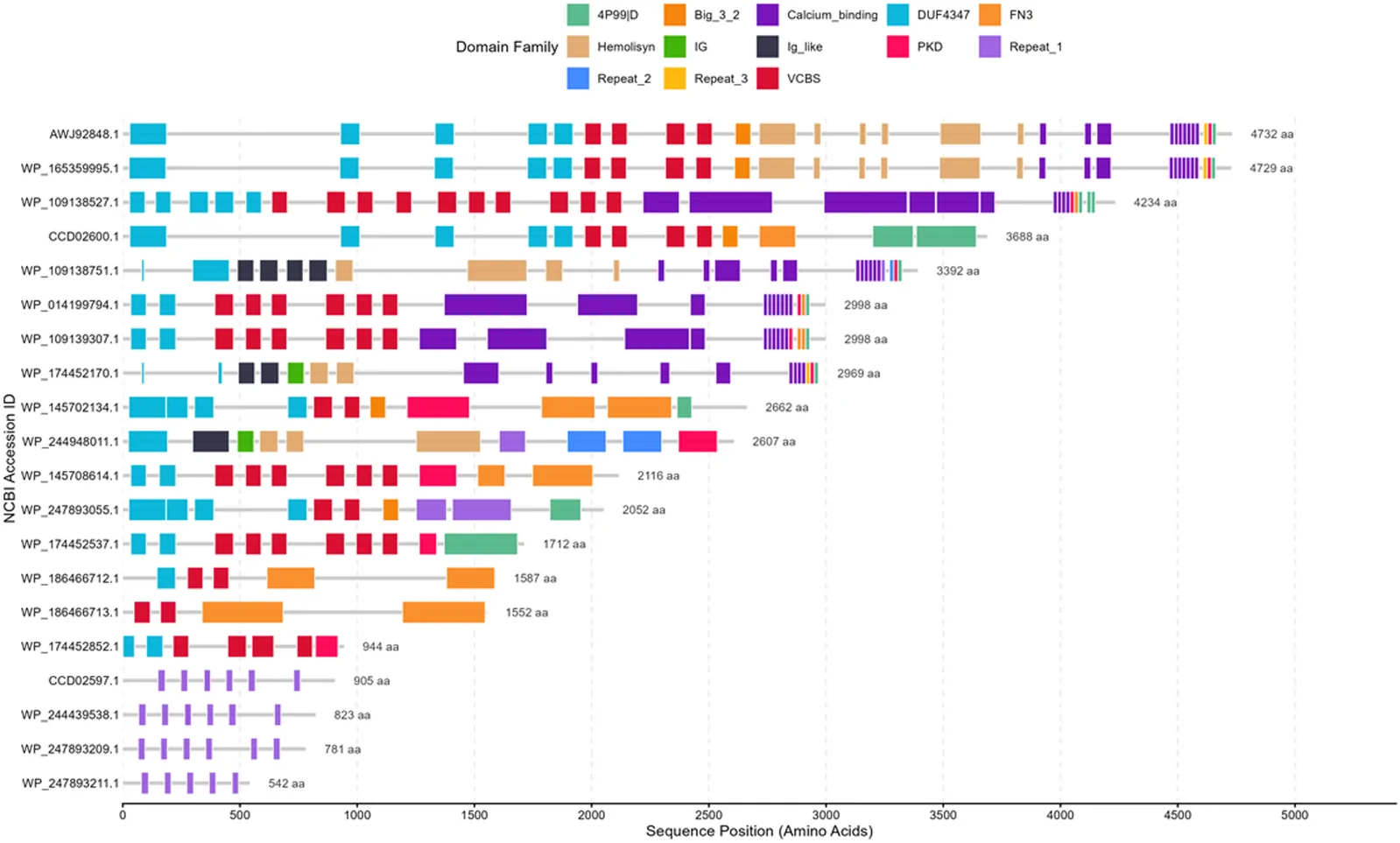
Predicted modular architectures of 20 Bap-like matrix protein candidates in *Azospirillum baldaniorum* Sp245. Schematic representation of the predicted modular architectures for 20 candidate protein sequences, ordered by total amino acid length. Proteins are draw to scale, with their respective sequence lengths indicated on the right. Domain annotations and identified structural features including VCBS, Ig-like, DUF4347, hemolysin-type calcium-binding, PKD, FN3, and related repetitive features, are color-coded according to the legend. Regions annotated by the SMART platform as hemolysin-type calcium-binding domains were further examined for characteristic calcium-binding nonapeptide repeats and associated hemolysin-related sequence features; these were classified as distinct structural motifs within the broader annotated region rather than as independent functional domains. Similarly, SMART-derived structural reference annotations (e.g., 4P99|D) correspond to specific Protein Data Bank (PDB) entries and chains, representing structural homology matches rather than discrete protein domains. Domain boundaries and remote homologies were further validated using HMMER3/Pfam and HHpred, while AlphaFold3 was utilized to predict and assess the overall three-dimensional structural organization of the candidate proteins. Data integration and graphical visualization were performed in R software using the **tidyverse** and **ggplot2** packages

In the absence of the curli machinery, we hypothesized that the strain utilizes alternative extracellular matrix proteins specifically those harboring amyloidogenic segments or RTX β-roll-forming motifs, which frequently facilitate calcium-dependent adhesion (Vance et al. 2014). To analyze this hypothesis, the *S. aureus* V329 Bap protein (AAK38834.2) was used as a reference sequence to screen for and identify candidate proteins encoded in the *A. baldaniorum* Sp245 genome that are potentially involved in biofilm matrix formation (Figure S1, Table 1, and Fig. 7).

**Fig. 8:**
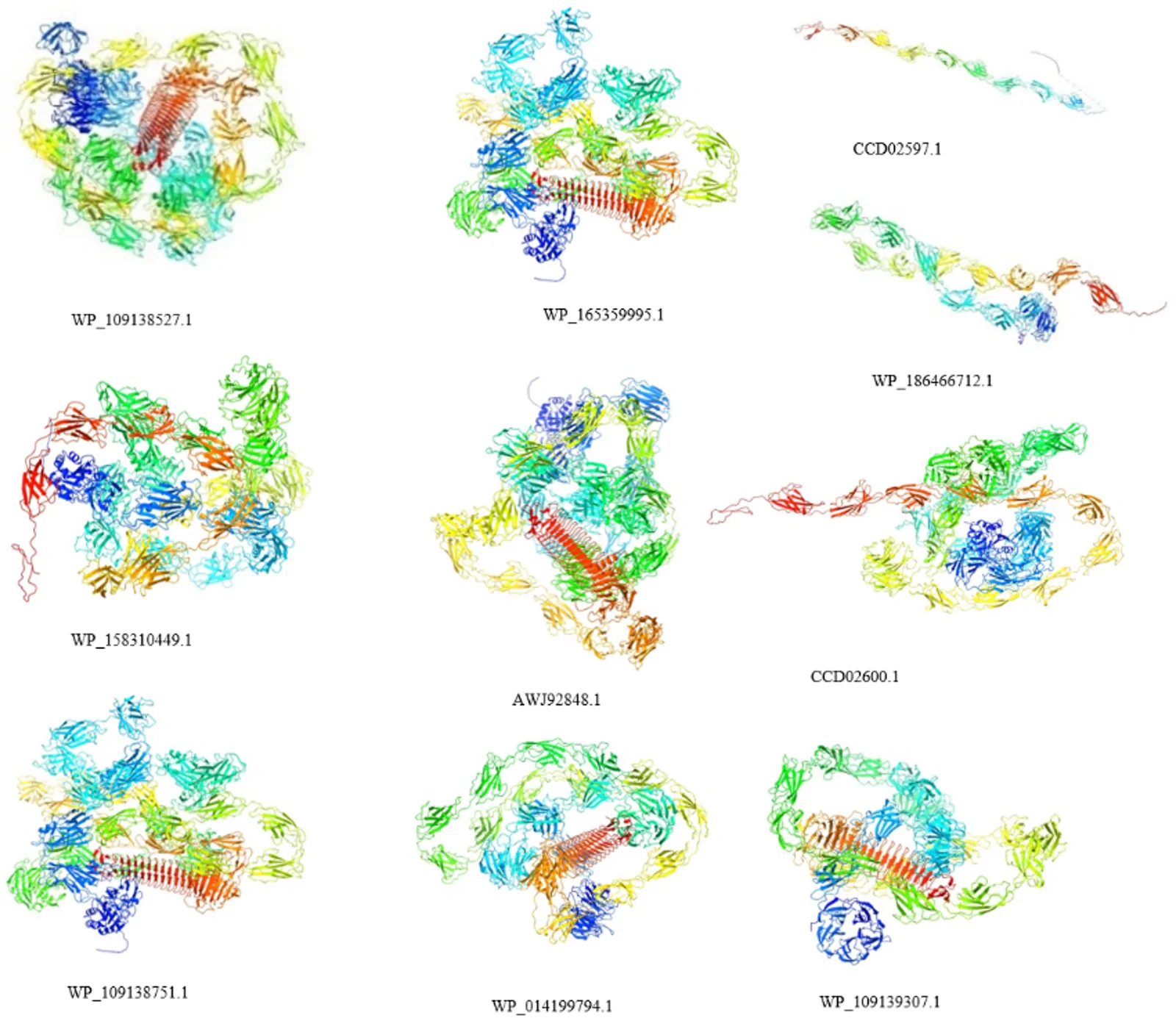
Predicted three-dimensional structures of representative Bap-like candidate proteins from *Azospirillum baldaniorum* Sp245. Representative AlphaFold3-predicted three-dimensional models of selected candidate proteins identified through the Bap-based sequence search. Full-length models are presented as ribbon representations using a rainbow color scheme, which maps the polypeptide chain sequentially from the N-terminal (blue) to the C-terminal (red) to visualize spatial progression along the linear sequence, with the corresponding NCBI protein accession indicated below each model. These models highlight the structural diversity observed among the candidate protein repertoire, providing a structural foundation for subsequent domain architecture analyses

**Fig. 9:**
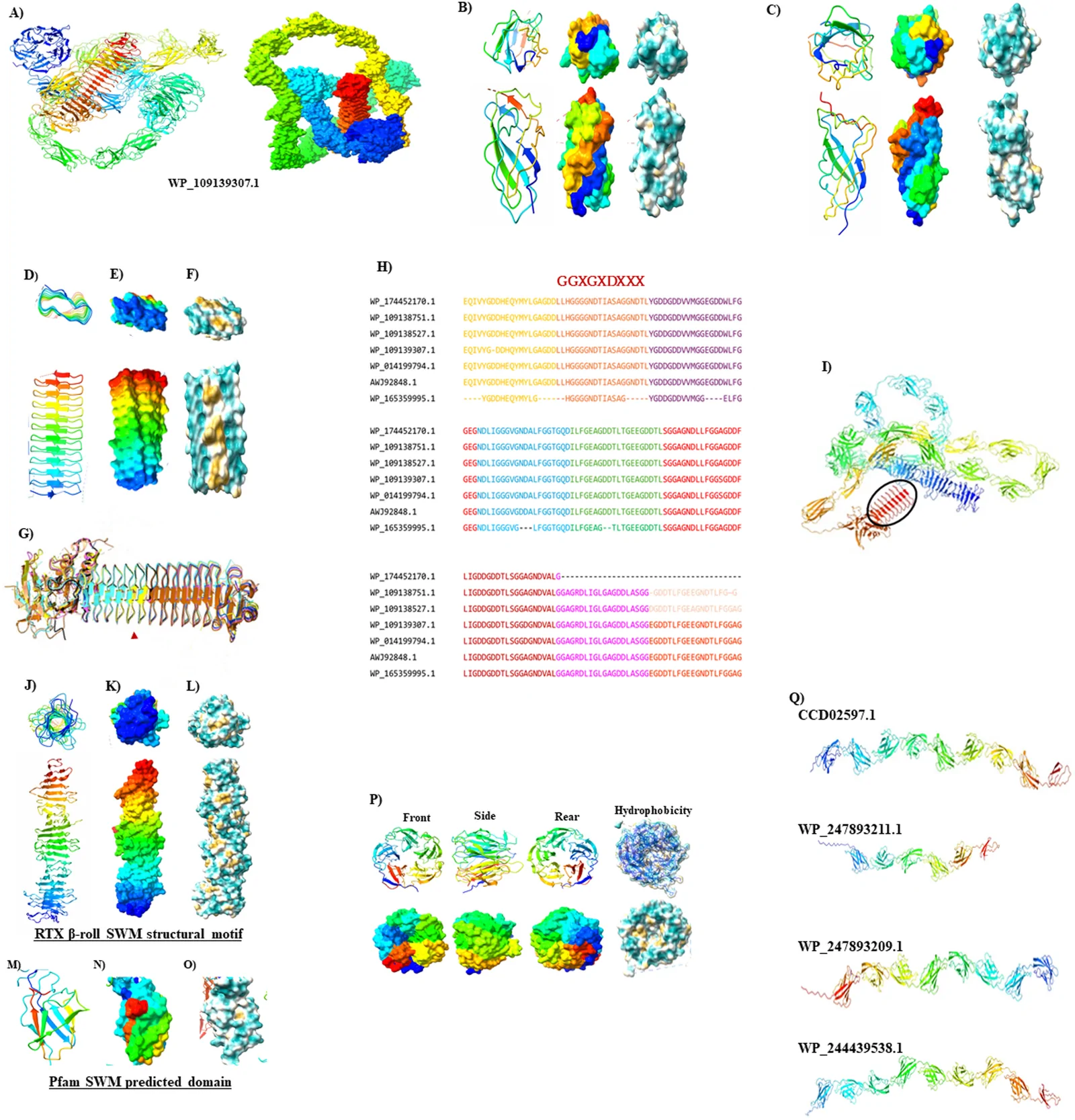
Structural and molecular features of functional domains and structural motifs in Bap-based candidate proteins from *Azospirillum baldaniorum* Sp245. **A** Predicted ribbon and surface structural models of a representative Bap-like protein (WP_109139307.1) from *A. baldaniorum* Sp245. This model illustrates the structural domain diversity observed across the analyzed protein set, with features consistent with previously reported amyloidogenic properties. Shown different views of *A. baldaniorum* Bap-like protein. **B** and **C** Structural and molecular analysis of Ig and Ig-like domains, respectively, extracted from the full-length protein WP_244948011.1. For each domain (left to right): predicted 3D ribbon model, surface representation, and hydrophobicity map. **D** Structural analysis of RTX β-roll repeats. **E** Surface representation of the RTX β-roll domain, displayed from front and axial orientations. **F** Hydrophobicity surface representation. **G** A ChimeraX-generated structural alignment shows the predicted 3D conformations of the RTX β-roll repeats, with a red arrow indicating the location of the calcium-binding motifs. **H** Clustal Omega multiple sequence alignment of RTX β-roll-forming regions from seven candidate proteins (AWJ_92848.1, WP_174452170.1, WP_109138751.1, WP_109138527.1, WP_109139307.1, WP_014199794.1, and WP_165359995.1). Conserved calcium-binding GGXGXDXXX motifs and their sequence are shown. **I** Complete predicted structure of WP_174452170.1, which was used for further molecular and structural characterization of the RTX β-roll domain. Predicted structure and conserved features of the Serine, Tryptophan, and Methionine (SWM) structural motif. **J** Ribbon representation of the SWM motif predicted by AlphaFold3, showing a compact β-sandwich architecture composed of two antiparallel β-sheets (front and axial views). **K** Surface topology and binding groove. **L** Hydrophobicity map of the SWM motif, with a color scale ranging from dark cyan (most hydrophilic) and white, to dark golden (most lipophilic). **M**–**O** Ribbon and surface representations and hydrophobicity map of the predicted Pfam SWM domain. **P** Structure of a five-bladed VCBS (β-propeller) domain. Ribbon representation of the five-bladed VCBS β-propeller in front, side, and rear views. Each blade (1–5) is formed by a four-stranded β-meander; the blades are arranged radially to create a toroidal fold around a central channel. Blades are colored sequentially (blade 1: blue; 2: cyan; 3: green; 4: yellow; 5: orange) to emphasize the repeating architecture. β-strands are shown as arrows, and connecting loops as ribbons. Surface representation: a prominent cleft at the face of the propeller (typically the top or “open” face) is highlighted as the putative carbohydrate/ion recognition site. Hydrophobicity map of the VCBS motif, with a color scale ranging from dark cyan (most hydrophilic) and white, to dark golden (most lipophilic). **Q** Predicted fimbriae amyloid-like folds suggest adhesin-related functions in the analyzed proteins. Ribbon representations of predicted structures for fimbriae amyloid-type proteins identified in the *A. baldaniorum* Sp245 genome, which are predominantly composed of Ig-like and/or Ig domains arranged in tandem

A total of 20 putative proteins were identified, with lengths ranging from 542 to 4,732 amino acid residues (Table 1, and Fig. 7). Their domain architecture is highly diverse, featuring VCBS, Ig-like, SWM, DUF4347, BIg-3-2, Hemolysin Calcium-binding, PKD, and FN3 domains, as well as various amino acid repeats (Type 1, 2, or 3). Notably, these proteins exhibit extensive domain reiteration, containing between 5 and 22 repeats per sequence (Table 1, and Fig. 7). Many candidates display structural signatures associated with amyloid-like assembly, such as repetitive β-strand motifs and RTX repeat architectures (Figs. 7 and 8, and 9A), suggesting a potential role in biofilm matrix development and the establishment of the RDAR morphotype.

The predicted three-dimensional models revealed elongated, filament-like conformations (Fig. 9B). A prominent feature in several proteins is the bacterial immunoglobulin-like (BIg) fold, also known as the BID domain (Bacterial Ig-like domain). These modules consist of approximately 74–120 amino acids arranged in tandem β-sandwich motifs (Fig. 9B and C). These folds are frequently integrated with RTX repeats or β-roll scaffolds, which likely enhance the stability and physical reach of cell-surface complexes (Chatterjee et al. 2021).

Detailed analysis of the RTX β-roll architecture showed an antiparallel, sandwich-like arrangement of β-sheets interconnected through canonical calcium-binding motifs. Hydrophobicity profiling indicates a distribution where hydrophobic residues from a protected internal core, while polar residues are oriented toward the solvent-exposed surface. Furthermore, the conserved calcium-binding motif (GGXGXDXXX) was identified in seven of the predicted proteins (Fig. 9D-I).

The SWM domain surface features a shallow groove bordered by polar residues, specifically conserved serine and acidic side chains supported by a compact β-sandwich architecture. Despite sequence divergence from canonical RTX repeats, SWM modules adopt an RTX-like β-roll fold. AlphaFold models suggest that SWM β-rolls maintain the elongated parallel β-sheet scaffold typical of RTX domains but utilize clusters of acidic side chain and small hydrophobic residues to compensate for atypical sequence compositions (Fig. 9J-O). These acidic clusters are predicted to coordinate Ca^2+^ ions, a mechanism essential for the folding and secretion of RTX-family proteins(Linhartová et al. 2010).

Additionally, we identified proteins containing a β-propeller domain, a fold found across all kingdoms of life(Pereira and Lupas 2021). For example, protein WP-10913937.1 exhibits a five-blade VCBS β-propeller. Each blade is composed of a four-stranded β-meander, arranged radially to form a toroidal fold around a central channel (Fig. 9P). Finally, structural analysis of accessions CCD02597.1, WP_247893209.1, WP_247893211.1 and WP_244439538.1 suggests functions related to fimbriae adhesins and amyloid-associated proteins (Fig. 9Q).

## Discussion

In this work, we initiated the study of the capacity of *A. baldaniorum* Sp245 to form macrocolonies of the RDAR morphotype. While this biofilm phenotype is exceptionally well-documented in model enteric microorganisms such as *E. coli* and *Salmonella* (Römling et al. 2005; Serra and Hengge 2017), its characterization in a PGPR*A. baldaniorum* represents a significant expansion of the RDAR paradigm. We observed that *A. baldaniorum* Sp245 develops this highly structured morphotype when grown on solid, NaCl-free LB medium supplemented with CR or a combination of CR and CBB, at 30 °C for 5 days.

In classic enteric models, the RDAR phenotype on CR agar correlates with the co-expression of curli fimbriae and cellulose (Serra and Hengge 2017). However, *A. baldaniorum* macrocolonies exhibited distinct dye-dependent pigmentation: red with CR alone, and brown with the CR + CBB combination. This differential staining suggests a high abundance of extracellular acting in concert with exopolysaccharides to constitute ECM. Importantly, the direct addition of fluorophores CFW, TS, and IP to the culture medium did not disrupt macrocolony topography. Utilizing the inverted agar method (Hickey and Read 2009) combined with CLSM, we visualized the presence of exopolysaccharides, eDNA, and probable amyloid proteins within the ECM. The concerted interaction of these three structural components is likely fundamental to maintaining the architectural integrity and mechanical recalcitrance of the biofilm lifestyle.

Previous characterizations on *Azospirillum* biofilms primary conducted at liquid-solid interfaces (plastic and glass) established that glucans, lipopolysaccharide complexes, and specific proteins act as an intercellular scaffold (Shelud’ko et al. 2018; Viruega-Góngora et al. 2020). For example, the presence of the Wheat Germ Agglutinin (WGA) hapten in the ECM highlights the critical role of lectin-carbohydrate interactions in anchoring mature biofilms to wheat root surfaces (Shelud’ko et al. 2018). However, our macrocolony models provide compelling evidence for an additional, highly resilient structural component: functional amyloid proteins.

Preliminary indirect evidence of amyloid structures in *Azospirillum* was previously noted via polarization microscopy, which revealed the hallmark yellow-green birefringence of CR-stained protein complexes (Shelud’ko et al. 2018). Because the interaction of cross-β protein structures with CR uniquely modifies the dye´s absorption spectrum, this birefringence is a good criterion for identifying amyloid fibrils in vitro and in vivo (Nilsson 2004). Functional amyloids such as curli, TasA, Bap, and Fap are ubiquitous across bacterial taxa and act as primary structural scaffolds in diverse biofilms (Fowler et al. 2007). In the current study, TS fluorescence and the presence of highly recalcitrant, formic-acid- resistant proteins detected in ECM support the presence of amyloid-like proteins in the *A. baldaniorum* RDAR biofilm matrix. However, genomic screening reported that *A. baldaniorum* lacks the *csgA* and *csgG* genes, indicating the absence of a canonical curli system. To elucidate the molecular basis of this curli-independent amyloidogenesis, we performed an in silico genomic analysis to identify alternative proteins with amyloidogenic and scaffolding properties, whose functions are described below.

Predicted Ig-like (BIg) folds are widely distributed in Gram-negative bacteria and are often found in large modular proteins associated with Type I and Type V secretion systems, where they mediate protein-protein interactions and contribute to adhesion(Chatterjee et al. 2021). In *A. baldaniorum*, predicted BIg, BIg-3-2, and Ig-like domains likely facilitate biofilm establishment by interacting with exopolysaccharides or plant root surfaces functioning as analogs to adhesion systems in other bacteria despite the absence of canonical curli operons. The coexistence of BIg/BID domains, Ig-like folds, and RTX-associated motifs suggests that *A. baldaniorum* utilizes a diverse, domain-rich repertoire to assemble its extracellular matrix.

### Predicted proteins harboring the VCBS structural domain

VCBS domains participate in diverse biological processes ranging from adhesion to transcriptional regulation(Fülöp and Jones 1999; Guruprasad and Dhamayanthi 2004). The β-propeller structure in these proteins typically serves as a recognition site for biomolecules, though it may also possess catalytic activity. For example, the five-blade tachylectin-2-like domain identified in WP_109139307.1 resembles eukaryotic lectins involved in innate immune responses (Weis and Drickamer 1996). The presence of VCBS repeats suggests that calcium-binding domains may contribute to the formation of amyloid-like structures and architectural stability within the biofilm.

### Predicted proteins harboring the RTX β-roll structural domain

RTX (Repeats in Toxin) proteins are prevalent in Gram-negative bacteria(Sakiyama et al. 2006). They are characterized by glycine-aspartate-rich repeats, typically located in the C-terminal region, which functions as calcium-binding motifs (Vance et al. 2014), and by their secretion via the Type I secretion system (Chenal et al. 2009). The putative RTX proteins identified in *A. baldaniorum* Sp245 likely contribute to extracellular matrix organization, adhesion, and bacteria-host interactions, underscoring their role in the biofilm´s structural architecture.

### Predicted proteins containing SWM structural motifs

The SWM (Serine-Tryptophan-Methionine) motif is a conserved module associated with surface-exposed and adhesion related proteins (McCarren and Brahamsha 2007), large proteins containing SWM motifs are often associated with swimming systems and biofilm formation (Linhartová et al. 2010), acting as modular units that facilitate cell surface interactions and matrix stabilization. Notably, the predicted protein WP_174452170.1 contains an SWM-like β-roll region despite lacking detectable homology to the canonical SWM Pfam domain. This highlights the distinction between sequence based and structure-based annotations: while Pfam identifies conserved sequence signatures, the SWM motif represents a β-roll architecture that can emerge from diverse sequences, particularly in glycine and acidic residues rich regions typical of RTX-like folds. AlphaFold models suggest that WP_174452170.1 forms a stable β-roll, even in the absence of tryptophan, indicating structural convergence. The presence of these diverse architectures suggests an evolutionary adaptation for survival in changing environmental conditions.

### Predicted proteins containing the DUF4347 domain

Domains of Unknown function (DUFs) represent a significant portion of the Pfam database. The DUF4347 domain, approximately 100 amino acids long, is typically located in the C-terminal or central regions of proteins in Proteobacteria, such as *Pseudomonas*, *Azospirillum*, *Burkholderia*, and *Xanthomonas*. Structural predictions indicate a β-sheet-rich globular fold, sometimes including β-roll or solenoid-like repeats. These domains are often associated with RTX repeats or calcium-binding motifs and are frequently located near genes encoding adhesins, secretion systems, or biofilm regulators(Bateman et al. 2010; Feng et al. 2024), suggesting a role in cell surface interaction or structural stability.

### Predicted proteins containing the PKD domain

Several identified proteins contain domains belonging to the Polycystic Kidney Disease (PKD) superfamily. Structurally, PKD domains consist of an eight-stranded β-sandwich fold, topologically similar to the 12 subsets of the immunoglobulin superfamily (IgSF) and fibronectin type III (FN3) superfamily (Bycroft et al. 1999). These domains are implicated in cell adhesion, mechanosensing, and biofilm-associated interactions across diverse organisms (Halaby and Mornon 1998). The *A. baldaniorum* Sp245 genome contains eight genes encoding putative PKD domains with 100% identity (Figure S5).

### Predicted fimbria amyloid-like proteins

Proteins such as intimin and invasin from *E. coli* (EPEC/EHEC), *Yersinia*, and *Salmonella* are known to mediate host adhesion via fibrillar, globin-related β-strand repeat structures (Oberhettinger et al. 2012). The predicted structures of *A. baldaniorum* proteins CCD02597.1, WP_244439538.1, WP_247893211.1 and WP_247893209.1 share topological similarities with intimin/invasin, though they lack strict sequence level motif conservation. Instead, they feature repeating β-strand units that form thin, rod-shaped, aggregative fimbria-like structures similar to those reported in *Enterobacteriaceae*.

## Conclusion

In conclusion, this study demonstrates that the PGPR *A. baldaniorum* Sp245 successfully develops the RDAR macrocolony morphotype in the air-solid interface (agar medium). Microscopic analyses suggest that the structural integrity of this biofilms relies on a complex extracellular matrix composed of exopolysaccharides, eDNA, and putative amyloid proteins. While direct experimental validation is currently constrained by the extensive diversity of these candidate genes and the technical challenges associated with classical genetic knockouts and antibody-based detection, we provide compelling preliminary evidence supporting the presence of amyloid-like structures. Furthermore, our computational analysis reveals a complex landscape of conserved amyloidogenic motifs, including Bap-like sequences, Ig-like domains, VCBS domains, SWM repeats, and RTX β-roll structures. These features are strongly associated with biofilm stability in other model microorganisms, suggesting a functionally conserved role for these structures in *A. baldaniorum*. Future research should prioritize the identification and functional characterization of these proteins and expanded genomic analysis to confirm their roles in biofilm assembly. Ultimately, a deeper understanding of these amyloid-related components could offer insights into plant-microbe interactions and facilitate the development of more effective bioinoculants for sustainable agriculture.

**Table 1:** The 20 Bap-based predicted high-molecular-weight candidate proteins with modular architectures potentially compatible with surface-associated or extracellular functions

Protein ID (NCBI)	Length (aa)	Description	SMART Predicted Domain Architecture (Counts)	Total Domains	Hotspots (**APPNN**)
WP_247893211.1	542	β-strand repeat	Big-3-2 (5)	5	33
WP_247893209.1	781	β-strand repeat	Big-3-2 (6)	6	52
WP_244439538.1	823	β-strand repeat	Big-3-2 (7)	7	54
CCD02597.1	905	Unclassified	Big-3-2 (7)	7	56
WP_174452852.1	944	Unclassified	VCBS (4), SWM (2), Hemolysin (Ca^+ 2^)-binding (1)	7	42
WP_186466713.1	1552	β-strand repeat	VCBS (2), Repeat 3 (2)	4	97
WP_186466712.1	1587	β-strand repeat	VCBS (2), SWM (1), Repeat 3 (2)	5	100
WP_174452537.1	1712	β-strand repeat	VCBS (6), SWM (2), Hemolysin (Ca^+ 2^)-binding (1), FN3 (1)	10	91
WP_247893055.1	2052	Ig-like	VCBS (2), SWM (4), DUF4347 (1), Repeat 1 (2), Repeat 3 (1)	10	121
WP_145708614.1	2116	Ig-like	VCBS (6), SWM (2), Hemolysin text (Ca^+ 2^) -binding (1), Repeat 2 (2)	11	121
WP_244948011.1	2607	β-strand repeat	Ig-like (1), SWM (1), Big-3-2 (3), Hemolysin (Ca^+ 2^)-binding (1), PKD (1), IG (1), Repeat 1 (2)	10	146
WP_145702134.1	2662	Ig-like	VCBS (2), SWM (4), DUF4347 (1), Hemolysin (Ca^+ 2^)-binding (1), Repeat 1 (2), Repeat 3 (1)	11	153
WP_174452170.1	2969	Ig-like	Ig-like (2), SWM (2), Big-3-2 (2), Hemolysin (Ca^+ 2^)-binding (9), PKD (1), IG (1), Repeat 1 (1), Repeat 3 (1)	19	153
WP_109139307.1	2998	Ig-like	VCBS (6), SWM (2), Hemolysin (Ca^+ 2^)-binding (10), PKD (1), Repeat 2 (2), FN3 (1)	22	165
WP_014199794.1	2998	Ig-like	VCBS (6), SWM (2), Hemolysin (Ca^+ 2^)-binding (10), PKD (1), Repeat 2 (1), FN3 (1)	21	165
WP_109138751.1	3392	Ig-like	Ig-like (4), SWM (2), Big-3-2 (4), Hemolysin (Ca^+ 2^)-binding (11), PKD (1), IG (1), Repeat 1 (1), Repeat 3 (1)	25	182
CCD02600.1	3688	Unclassified	VCBS (4), SWM (5), DUF4347 (1), Repeat 1 (1), Repeat 2 (2)	13	199
WP_109138527.1	4234	Ig-like	VCBS (10), SWM (5), Hemolysin (Ca^+ 2^ )-binding (10), PKD (1), Repeat 2 (1), Repeat 3 (1), FN3 (3)	31	238
WP_165359995.1	4729	Ig-like	VCBS (4), SWM (5), DUF4347 (1), Big-3-2 (7), Hemolysin Ca^+ 2^ )-binding (10), PKD (1), Repeat 1 (1), Repeat 3 (1)	30	254
AWJ92848.1	4732	Unclassified	VCBS (4), SWM (5), DUF4347 (1), Big-3-2 (7), Hemolysin Ca^+ 2^)-binding (10), PKD (1), Repeat 1 (1), Repeat 3 (1)	30	254

Number of amyloid hotspots predicted by APPNN (Amyloid Propensity Prediction Neural Network) Familia C et al. 2015

^a^ Sequence alignment parameters derived from local BLASTp searches using Staphylococcus aureus V329 Bap (AAK38834.2) as the query

^b^ aa, amino acids; Big, bacterial immunoglobulin-like domain; DUF, domain of unknown function; FN3, fibronectin type III domain; IG, immunoglobulin-like domain; PKD, polycystic kidney disease domain; SWM, SwmM-like repeat; VCBS, Vibrio, Colwellia, Bradyrhizobium, and Shewanella domain

^c^ The table is ordered according to increasing predicted length-structural complexity to facilitate comparison and candidate prioritization for future experimental studies

## Supplementary Information

Below is the link to the electronic supplementary material.

Supplementary Material 1

## Data Availability

No datasets were generated or analysed during the current study.
