# Supplementary material for: RDAR biofilm formation and in silico analysis of putative amyloid proteins in Azospirillum baldaniorum Sp245
Source: Arch Microbiol. 2026 Oct 31;209(1):42. doi: 10.1007/s00203-026-05297-4 (PMC13723633; doi:10.1007/s00203-026-05297-4)
Supplement: Supplementary file 1 [file 203_2026_5297_MOESM1_ESM.docx]

**Supplementary Information (SI)**

**RDAR Biofilm Formation and In Silico Analysis of Putative Amyloid Proteins in *Azospirillum baldaniorum* Sp245**

**Figure S1 Predicted modular architecture, domain composition, and structural models of functional amyloids proteins.**

The structural organization of the *Escherichia coli* curli fimbriae subunit (GenBank: CAD601748) and the *Staphylococcus aureus* V329 Bap protein (GenBank: AAK38834.2) are shown. A) Domain architecture predicted via the SMART platform and the corresponding AlphaFold structural for *E. coli* CAD601748. B) Domain architecture predicted via the SMART platform and the corresponding AlphaFold structural model for *S. aureus* V329 AAK38834.2.

**Figure S2. Analysis of the *A. baldaniorum* Sp245 genome for Curli fimbriae (CsgA) homologs.** BLASTP analysis using the CsgA amino acid sequence revealed no significant similarity to any open reading frames within the *A. baldaniorum* Sp245 genome.

**Figure S3. Analysis of the *A. baldaniorum* sp245 genome for Curli channel (CsgG) homologs.** BLASTP analysis using the CsgG amino acid sequence revealed no significant similarity to any open reading frames within the *A. baldaniorum* Sp245 genome.

**Figure S4. Identification of putative Bap-like proteins in the *A. baldaniorum* Sp245 genome.** BLASP analysis was performed using the amino acid sequences of the *Staphylococcus aureus* V329 Bap protein (GenBank: AAK38834.2) as a query to identify homologous sequences within the *A. baldaniorum* Sp245 genome.

**Figure S5.** Amino acid alignments of the PKD domain found in the proteins analyzed in this study.

**Video 1 High-resolution timelapse visualization of the *A. baldaniorum* Sp245 RDAR-type biofilm.**

Wild-type strain macrocolonies were grown on LB* agar for 5 days at 30°C. CLSM images were acquired using a 60x objective from the central zone of the biofilm, with 1.5 digital zoom. Data are representative of three independent experiments with three technical replicates. 3D projections depict the spatial distribution of biofilm components stained with fluorescent dyes: CFW, exopolysaccharides (blue); TS, amyloids (green); and PI, eDNA (red).
